# Felbamate for pediatric epilepsy—should we keep on using it as the last resort?

**DOI:** 10.3389/fneur.2022.979725

**Published:** 2022-09-20

**Authors:** Shira Rabinowicz, Tal Schreiber, Gali Heimer, Omer Bar-Yosef, Andreea Nissenkorn, Zohar-Dayan E, Leo Arkush, Nasrin Hamed, Bruria Ben-Zeev, Michal Tzadok

**Affiliations:** ^1^Pediatric Neurology Unit, Edmond and Lily Safra Children's Hospital, Sheba Medical Center, Ramat-Gan, Israel; ^2^Sackler School of Medicine, Tel-Aviv University, Tel-Aviv, Israel

**Keywords:** felbamate, epilepsy, electrical status epilepticus during sleep, herpes, drug resistance

## Abstract

**Introduction:**

Concerns regarding felbamate adverse effects restrict its widespread use in children with drug-resistant epilepsy. We aimed to examine the efficacy and safety of felbamate in those children and identify the ones who may benefit most from its use.

**Methods:**

We retrospectively reviewed the medical files of all patients who were treated with felbamate in a tertiary pediatric epilepsy clinic between 2009–2021. Drug efficacy was determined at the first 3 months of treatment and thereafter. Therapeutic response and adverse reactions were monitored throughout the course of treatment.

**Results:**

Our study included 75 children (age 8.9 ± 3.7 years), of whom 53 were treated with felbamate for seizures, 16 for electrical status epilepticus during sleep and 6 for both. The median follow-up time was 16 months (range 1–129 months). The most common cause for epilepsy was genetic (29%). The median number of previous anti-seizure medications was six [4–8]. A therapeutic response ≥50% was documented in 37 (51%) patients, and a complete response in 9 (12%). Nineteen patients (25%) sustained adverse reactions, including three cases of elevated liver enzymes and one case of neutropenia with normal bone marrow aspiration. In all cases, treatment could be continued. All children with intractable epilepsy following herpes encephalitis showed a response to felbamate.

**Conclusion:**

Felbamate is an efficacious and safe anti-seizure medication in the pediatric population.

## Introduction

Epilepsy is a common disease of childhood, affecting 0.5–1% of the pediatric population (1, 2). Anti-seizure medications (ASMs) are the mainstay of treatment. However, only 58–80% of epilepsy patients become seizure free with either the first- or second-line ASMs, and the others may be defined as having a “drug-resistant” epilepsy (2–6). Intractable epilepsy may have severe consequences, from psychosocial disabilities and educational underachievement which may impair quality of life, to mortality (7–9).

A dozen newer ASMs have been introduced in recent years, but therapeutic response rates were not dramatically modified (3). The ASM, felbamate, was shown to be effective in medical trials after having been first approved by the United Stated Food and Drug administration in 1993 as a monotherapy or an adjuvant therapy for the management of focal seizures and Lennox-Gastaut syndrome (LGS) (10), and then later for other types of seizures (11–13). Reports on adverse events emerged soon after its release, with aplastic anemia and liver failure being of greatest concern (11, 14). As a result, its use is currently limited to patients with refractory epilepsy following judicious consideration of the benefits and risks (10).

The suggested mechanisms of action of the drug are: antagonism of n-methyl-D-aspartate (NMDA) receptor, potentiation of γ-aminobutyric acid (GABA)-ergic activity and the inhibition of voltage-gated sodium and calcium channels (10, 15).

We aimed to examine the efficacy and safety of felbamate in children with drug-resistant epilepsy, including seizures and electrical status epilepticus during sleep (ESES), and to identify the ones who may benefit the most from its use.

## Methods

### Setting and subjects

This study is a retrospective medical chart review carried out in the Pediatric Neurology Unit, Safra Children's Hospital, Sheba Medical Center, Israel. The cohort includes all children up to 18 years old who began treatment with felbamate in our tertiary referral epilepsy clinic between 2009–2021. The study was approved by the institution's ethics committee.

### Data collection

The retrieved data included demographics, seizure types and frequency, epilepsy history, as well as previous and current treatments, including ASMs, immune modulating drugs (steroids and intravenous immunoglobulins), and cannabinoids. Also recorded were data on vagal nerve stimulation, the use of a ketogenic diet, the felbamate therapeutic dose, treatment response as assessed by the treating physician at routine visits, adverse events and blood tests results. We also reviewed the findings on the children's electroencephalograms (EEGs) before and during treatment with felbamate.

### Treatment regimen

The treatment regimen was a starting dose of 10 mg/kg/day, with gradual increase over one month up to 30–40 mg/kg/day to a maximal dose of 1,800 mg/day. Doses were changed as needed on an individual basis. Complete blood counts and chemistry, including liver enzymes, were taken prior to treatment, once during dose elevation, again at maximal dose, and then every 3 months during follow-up. Adverse reactions were monitored by reported symptoms and routine blood tests.

### Outcome measures

Response was defined as a ≥50% reduction in seizure frequency according to verbal reports by the family to the treating clinician in patients with clinical seizures, or a ≥50% reduction in spike and wave index (SWI) in EEG recordings among the ESES patients, measured within the first 3 months of treatment. Thereafter, the therapeutic response was reported during routine visits according to the same criteria. The last clinic visit was considered as the last date of ongoing felbamate treatment.

### Statistical analysis

Continuous variables were evaluated for normal distribution and summarized as mean and standard deviation (SD) or median and interquartile range (IQR). Categorical variables were reported as frequency and percentage. Continuous variables were compared by means of an independent samples *t*-test or a Mann Whitney test, and categorical variables were compared with the chi-square or Fisher's exact test. Kaplan-Meir curves were used to describe treatment discontinuation during the follow-up period. A reverse censoring method was used to evaluate the median length of follow-up. All statistical tests were two-sided, and a *p*-value <0.05 was considered statistically significant. Analyses were performed by SPSS software (IBM SPSS Statistics for Windows, Version 25.0. Armonk, NY: IBM Corp., 2017).

## Results

Our study included 75 children (46 boys and 29 girls, mean ± SD age 8.9 ± 3.7 years, range 2.8–17.4 years), of whom 53 were treated for seizures, 16 for ESES without clinical seizures and 6 for both indications. The median follow-up time was 16 months (range 1–129 months). The most common cause of epilepsy was genetic (Table 1), including patients with findings in chromosomal microarray tests or mutations in specific genes (CDKL5, TSC2, etc.). This was followed by epilepsy due to structural lesions (brain malformations, vascular or traumatic events), infection (post-herpes simplex virus encephalitis), immune reaction (Rasmussen encephalitis) and metabolic (post-hypoglycemia).

**Table 1:** Demographic data of responders and non-responders.

		**All children** **(*n* = 75)**	**Therapeutic response ≥50% (*n* = 37)**	**Therapeutic response <50% (*n* = 36)**	***p*–value**
Age at treatment initiation (years, mean, SD)		8.9 ± 3.7	9.3 ± 3.5	8.7 ± 3.9	0.44
Age at start of epilepsy (years, median, IQR)		2.75 (0.5–5)	3.6 (0.5–5)	2.9 (0.6–5.7)	0.98
Age <12 years, *n (%)*		57 (78)	29 (78)	28 (78)	>0.99
Male sex, *n (%)*		46 (61.3)	17 (46)	27 (75)	0.017
Time from epilepsy onset to treatment (years, median IQR)		4.5 (3.2–8.2)	5 (2.9–9.7)	4.2 (3.2–6.1)	0.5
Previous ASMs, n		6 (4–8)^b^	5 (4–7)	6 (5–7.75)	0.25
Previous use of immunotherapy, *n (%)*		28 (37.3)	15 (53.6)	13 (46.4)	0.81
Previous use of CBD, *n (%)*		16 (21.3)	10 (62.5)	6 (37.5)	0.4
Prior ketogenic diet, *n (%)*		23 (30.6)	12 (54.5)	10 (45.5)	0.8
Prior VNS, *n (%)*		12 (16)	6 (50)	6 (50)	>0.99
Concurrent VNS, *n (%)*		6 (8)			
Prior epilepsy surgery, *n (%)*		2 (2.6)	1 (50)	1 (50)	>0.99
Etiology, *n*	Structural (%)	20 (26.6)	9 (45)	11 (55)	0.61
	Genetic (%)	22 (29.3)	12 (57.1)	9 (42.9)	0.61
	Infectious (%)	4 (5.3)	4 (100)	0	0.06
	Immune (%)	1 (1.3)	1 (2.7)	0	0.5
	Metabolic (%)	2 (2.6)	1 (50)	1 (50)	>0.99
	Unknow*n (%)*	26 (34.6)	10 (40)	15 (60)	0.22

SD, standard deviation; IQR, interquartile range; ASM anti–seizure medication; CBD, cannabidiol; VNS, vagal nerve stimulation.

**Figure 1:**
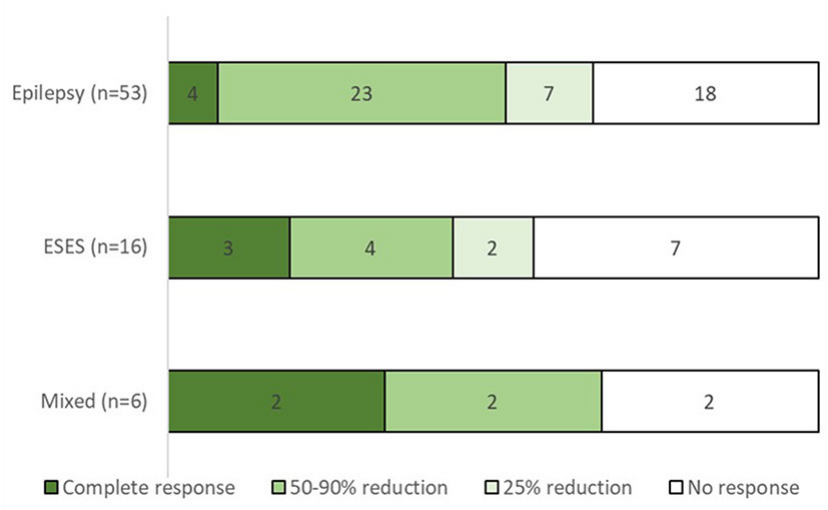
Response rate by treatment indication.

All the study patients had drug resistant epilepsy; The median number of previous ASMs was six (IQR 4–8), and 32 patients (43%) had undergone at least one non-pharmacological therapy, such as vagal nerve stimulation, ketogenic diet and epilepsy surgery (Table 1). The vast majority of the study patients (73/75) were treated with ASMs concurrently with felbamate. Two patients were subsequently excluded from the response analysis: one had started on felbamate 20 mg/kg following status epilepticus and discontinued it after a few days due to urticaria, and the other was lost to follow-up. A response rate ≥50% was documented in 37 of the remaining 73 (51%) patients (Figure 1). Six of the patients treated for seizures became seizure free (two of the six were also treated for ESES). Three patients experienced seizure exacerbation. Six patients underwent vagus nerve stimulator (VNS) implantation during treatment with felbamate.

Our cohort included 22 patients with ESES, of whom 6 had clinical seizures in addition. Their median age was 8.8 years (IQR 7.2–11.1 years). 15/22 patients have failed immunologic treatments (all were treated with intravenous pulse methylprednisolone therapy and four patients received additional intravenous immunoglobulins).

**Figure 2:**
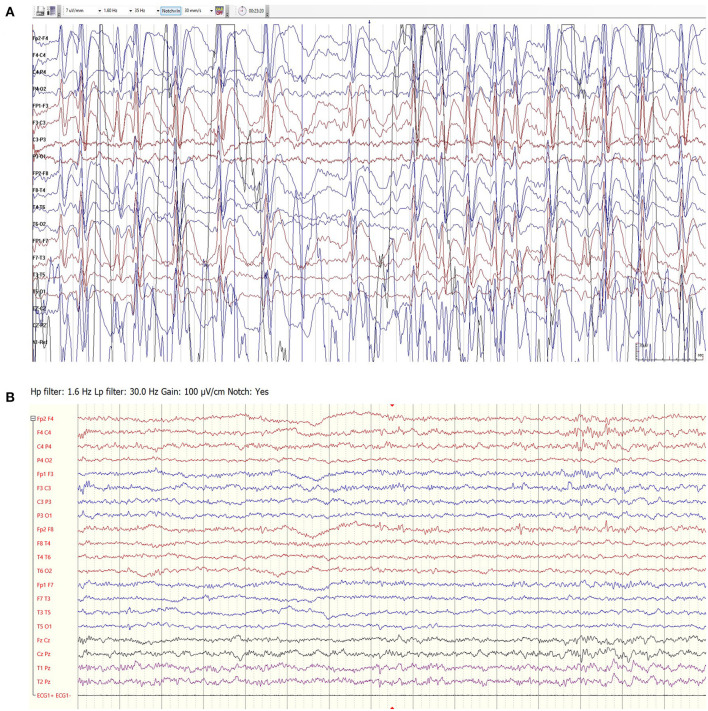
EEG recordings of a 7.5-year-old girl with idiopathic ESES prior to felbamate treatment **(A)** and following 3 months of treatment **(B)**. The response lasted 10 months.

Among the 16 patients treated only for the indication of ESES, the median course of felbamate lasted 195 days (IQR 97–441 days). Four patients had a complete response, meaning 100% reduction in spike and wave index (SWI) in EEG recordings following treatment. EEG recordings of a sample patient before and ^*^ months after treatment can be seen in Figure 2.

The cohort included seven patients with Lennox- Gastaut syndrome, of whom one had a transient response to felbamate that lasted 3 months. Three additional patients had a minimal response of 25% reduction in seizure frequency and were considered as non- responders. The remaining three patients discontinued treatment after a few weeks due to no efficacy.

Thirty-five of the 75 study patients discontinued felbamate treatment due to lack of efficacy (24 patients), aggravation of seizures (seven patients) and side effects (four patients).

**Figure 3:**
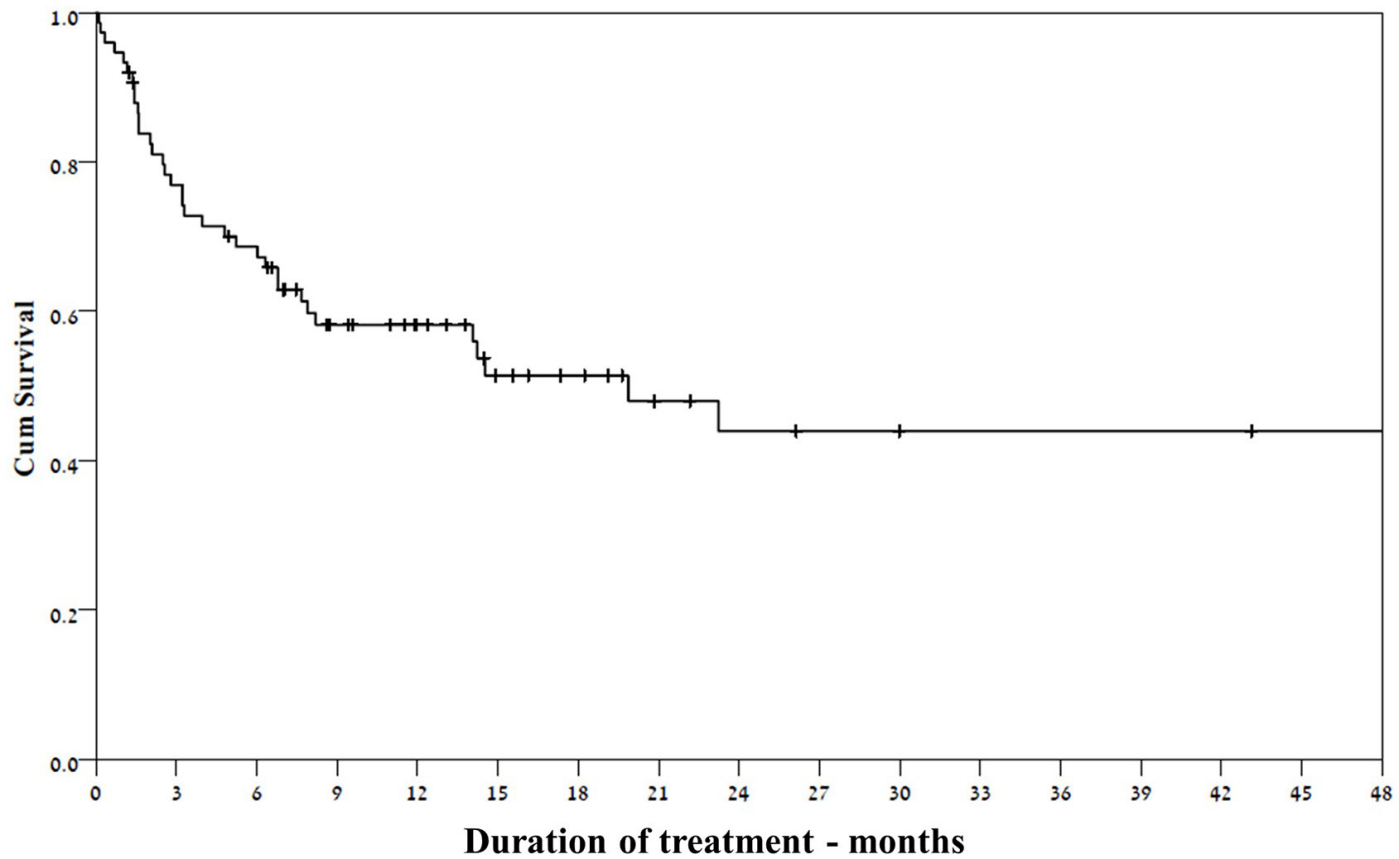
Kaplan- Meier curve of patients continuing treatment with felbamate over time.

Thirty-one of the 37 patients who responded at 3 months continued the treatment until the end of follow- up (Figure 3), despite waning efficacy in five of them. The response rate among girls was significantly higher than that for boys (69 vs. 38.6%, respectively, *p* = 0.017). Other selected factors were not associated with response to felbamate (Table 1).

The response to the drug among a few patients may be attributed to the mechanism of their disease. One was a 4-year-old girl with intractable daily bilateral focal seizures, who had been previously treated with eight ASMs, a ketogenic diet and cannabidiol. Her magnetic resonance imaging study was normal, and her genetic work-up revealed a heterozygote (c.357G>T) variant of uncertain (VUS) significance in the ATP1A2 gene, in addition to VUS in the PRICKLE1 gene. she became seizure free on felbamate 40 mg/kg. She underwent a bone marrow biopsy due to a decrease in her neutrophil count (from 1230 cells/mm^3^ to 530 cells/ mm^3^) whose results showed normal cellularity. She continued treatment with a reduced dose of felbamate (20 mg/kg/day) and remained seizure free for two years. Another patient was a 12-year-old girl heterozygote to a GRIN2B mutation (c.1664 G>C). Her phenotype included severe intellectual disability and various types of resistant seizures. The addition of felbamate (30 mg/kg) to levetiracetam, clobazam, lamotrigine and cannabidiol resulted in complete remission of seizures that lasted 1 year and had never occurred before with other ASMs.

**Figure 4:**
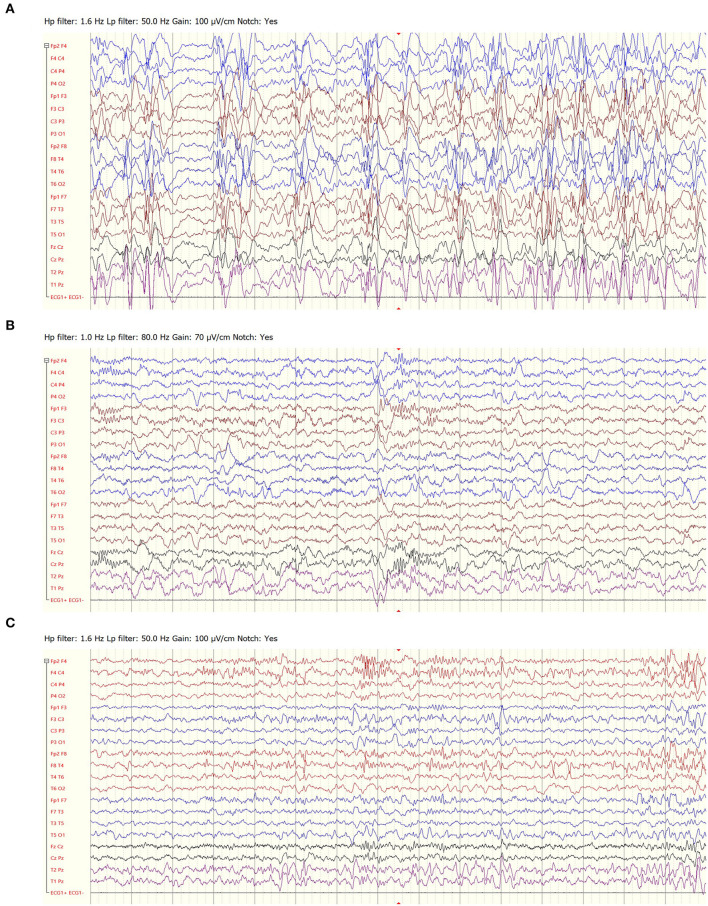
EEG recordings of a 4-year-old girl with epilepsy following herpes encephalitis, prior to felbamate treatment **(A)**, following 3 months of treatment **(B)** and following 3 years of treatment **(C)**.

Four patients in our cohort suffered from epilepsy following infection with herpes encephalitis. All patients had an initial clinical response. The response lasted a year in three patients, who later had a phenotype consistent with LGS. Another patient, whose phenotype included both clinical seizures and ESES, became seizure free and had a marked improvement of the EEG, lasting more than 3 years (Figure 4)

Adverse reactions to felbamate were reported in 19/75 patients (Table 2). Five of them required dose reduction and five others stopped treatment. The median age of the patients who experienced side effects was 7.5 years (IQR 4.3–10.3). All patients with adverse events were treated with concurrent medications, the most common were clobazam (9 patients), valproic acid (7) and phenobarbital (5).

**Table 2:** Prevalence of adverse events in 75 patients treated with felbamate.

**Sign/Symptom**	**Number of patients**
Elevated liver enzymes	3
Fatigue	2
Behavioral symptoms	2
Ataxia	2
Allergic response	1
General deterioration	1
Neutropenia	1
Salivation	1
Urinary incontinence	1
Loss of appetite	1
Insomnia	1
Constipation	1
Dizziness	1

None of our patients experienced liver failure or aplastic anemia. Three patients had elevated liver enzymes: aminotransferases up to twice the upper normal limit and γ-glutamyl transferase (GGT) up to four times the upper normal limit. One of them started felbamate concurrently with valproic acid, but liver enzymes increased only a few months after discontinuation of valproic acid. The neutrophil count decreased in one patient.

## Discussion

This is the largest cohort of children with various epileptic syndromes who were treated with felbamate, a drug with unique mechanisms of action among the variety of different currently available ASMs (16, 17). Our findings show that one-half of pediatric patients with drug resistant epilepsy responded to add-on felbamate, with a reasonable rate of side effects and no life-threatening adverse events. This is consistent with previous findings in smaller groups of children (12, 13, 18) and adults (18) treated with felbamate. The rate of responders among resistant patients following a median of six previous drugs was higher than expected (5). Furthermore, we observed a durable response in a drug resistant population, as roughly 70% of the patients who initially responded to felbamate maintained response until the end of their follow-up.

The spectrum of side effects was similar to previously reported data. We witnessed three cases of elevated liver enzymes, but decided to cautiously continue treatment in those patients, of whom two continued follow up with no further deterioration. Also, despite major concerns regarding aplastic anemia in the patient with worsening neutropenia, a normal bone marrow biopsy enabled us to continue felbamate treatment in a girl with debilitating epilepsy, suggesting that doing so under careful supervision is apparently not detrimental. Dozières-Puyravel et al. had reported reversible neutropenia children treated with felbamate (19). Additionally, Zupanc et al. (20) described safe treatment with felbamate in patients with a history of bone marrow disorders, following harvesting of stem cells for use in case of aplastic anemia. We acknowledge the fact that our cohort may have been too small to screen for rare and fatal adverse events. However, we can suggest that felbamate is generally safe and should be considered for the treatment of refractory epilepsy in the pediatric population.

The association between female sex and response to felbamate was statistically significant in the univariate analysis. This has not been previously reported and there is no apparent mechanism. Further investigation is warranted to determine whether this finding is relevant.

We observed a remarkable response in a few patients whose genetic diagnosis may be linked to the mechanism of action of the drug. The response of a patient with a GRIN2B mutation raises the possibility of a gain of function mutation in response to NMDA receptor inhibition (20). The response in the case of a child with a variant in the ATP2A1 gene, which encodes the α-2 subunit of Na+/K+ ATPase pump in glial cells, may be explained by the coupling of the ATPase and the excitatory amino acid transporter, leading to excess of glutamate in the synaptic cleft (21, 22). However, the variant was categorized as a VUS and was not checked for inheritance or obvious pathogenicity.

Notably, children with epilepsy following herpes encephalitis showed a good response to felbamate treatment. This observation suggested that there might be a connection between the pathophysiology of herpes-induced epilepsy and response to felbamate. It has been hypothesized that herpes simplex virus-mediated brain injury leads to exposure of NMDAR and the development of anti-NMDAR autoimmune encephalitis (23). This may theoretically explain the response to felbamate, an NMDA receptor blocker, and further investigation of this mechanism is warranted.

Reports on dramatic EEG improvements following treatment with felbamate in patients with ESES are scarce, and they include ESES as part of Lennox-Gastaut syndrome (12) and a few case reports (24, 25). Interestingly, six of seven patients diagnosed with Lennox- Gastaut syndrome as the original indication for the drug did not respond (12). Two of them received felbamate as a 4th drug (of whom one responded), while the others were offered felbamate later in the disease course. This was in part because many patients only fulfilled diagnostic criteria for LGS after a period of time being treated empirically for epilepsy using more conventional ASMs. Another factor may be that our clinic serves as a tertiary epilepsy referral center so some patients were managed elsewhere. Nonetheless, those findings suggest that felbamate may achieve higher response rate in populations other than its original indication.

The main limitation of our study is its retrospective design, as a result of which evaluation and follow-up were done for clinical rather than investigational purposes. Moreover, other interventions had taken place concomitantly (for example, VNS implantations during treatment with felbamate might have affected the response analysis of six patients). Felbamate levels were not measured, since this was not the standard of care. Finally, our population consisted of drug resistant patients referred to a specialized epilepsy clinic.

In conclusion, we report good efficacy and safety of felbamate in the largest cohort of children with different types of drug-resistant epilepsy treated with felbamate in our tertiary pediatric epilepsy clinic.

## Data availability statement

The raw data supporting the conclusions of this article will be made available by the authors, without undue reservation.

## Ethics statement

The studies involving human participants were reviewed and approved by Sheba Medical Center Helsinki Commmittee. Written informed consent from the participants' legal guardian/next of kin was not required to participate in this study in accordance with the National Legislation and the Institutional Requirements.

## Author contributions

SR and TS: acquisition, analysis, interpretation of data, and drafting the manuscript. GH, OB-Y, AN, Z-DE, LA and NH: data acquisition and manuscript revision. BB-Z: design and conception of the work and manuscript revision. MT: design and conception of the work, data analysis, and manuscript revision. All authors contributed to the article and approved the submitted version.

## Conflict of interest

The authors declare that the research was conducted in the absence of any commercial or financial relationships that could be construed as a potential conflict of interest.

## Publisher's note

All claims expressed in this article are solely those of the authors and do not necessarily represent those of their affiliated organizations, or those of the publisher, the editors and the reviewers. Any product that may be evaluated in this article, or claim that may be made by its manufacturer, is not guaranteed or endorsed by the publisher.
